# Soluble Epidermal Growth Factor Receptors (sEGFRs) in Cancer: Biological Aspects and Clinical Relevance

**DOI:** 10.3390/ijms17040593

**Published:** 2016-04-19

**Authors:** Sally Maramotti, Massimiliano Paci, Gloria Manzotti, Cristian Rapicetta, Mila Gugnoni, Carla Galeone, Alfredo Cesario, Filippo Lococo

**Affiliations:** 1 Laboratory of Translational Research, Research and Statistic Infrastructure, Arcispedale Santa Maria Nuova-IRCCS, Reggio Emilia 42123, Italy; gloria.manzotti@asmn.re.it (G.M.); mila.gugnoni@asmn.re.it (M.G.); 2 Thoracic Surgery Unit, Department of Cardiology, Thoracic and Vascular Surgery, Arcispedale Santa Maria Nuova-IRCCS, Reggio Emilia 42123, Italy; massimiliano.paci@asmn.re.it (M.P.); cristian.rapicetta@asmn.re.it (C.R.); carla.galeone@asmn.re.it (C.G.); 3 Head, Systems Medicine, Arcispedale Santa Maria Nuova-IRCCS, Reggio Emilia 42123, Italy; alfredo.cesario@asmn.re.it

**Keywords:** soluble receptors, soluble epidermal growth factor receptor (sEGFR), circulating biomarkers

## Abstract

The identification of molecules that can reliably detect the presence of a tumor or predict its behavior is one of the biggest challenges of research in cancer biology. Biological fluids are intriguing mediums, containing many molecules that express the individual health status and, accordingly, may be useful in establishing the potential risk of cancer, defining differential diagnosis and prognosis, predicting the response to treatment, and monitoring the disease progression. The existence of circulating soluble growth factor receptors (sGFRs) deriving from their membrane counterparts has stimulated the interest of researchers to investigate the use of such molecules as potential cancer biomarkers. But what are the origins of circulating sGFRs? Are they naturally occurring molecules or tumor-derived products? Among these, the epidermal growth factor receptor (EGFR) is a cell-surface molecule significantly involved in cancer development and progression; it can be processed into biological active soluble isoforms (sEGFR). We have carried out an extensive review of the currently available literature on the sEGFRs and their mechanisms of regulation and biological function, with the intent to clarify the role of these molecules in cancer (and other pathological conditions) and, on the basis of the retrieved evidences, speculate about their potential use in the clinical setting.

## 1. Introduction

Soluble receptors were identified more than thirty years ago. The regulatory role of cytokines and growth factors as “molecular controllers” of the immune response, inflammatory reactions, cell proliferation, and tumor growth has been at the focus of research activities for a long time; the intensive study on these molecules has been accompanied along with the investigation of their interaction with specific cell-surface receptors. Cytokines and growth factors have relevant biological effects and are regulated at two major levels of control: (a) the production and secretion of soluble molecules (ligand) and (b) the expression and cell surface concentration of the target receptor. When the ligand engages with the exposed extracellular domain of the receptor, the binding triggers conformational changes on the receptor itself that, in turn, lead to the activation of a kinase cascade that transduces the signal from the outside of the cells to its nucleus. Soon after, the characterization of the membrane growth factor receptors, it was established that cells are able to also produce a soluble form of these receptors (sGFRs), containing only the extracellular portion that retains the ability to interact with the ligands but lacks the ability to activate the intracellular signaling cascade. The high affinity of soluble receptors for the ligands allows these soluble isoforms to compete with the full-length receptors thus reducing the activation of the growth factor dependent cascade [1,2]. The prototype and by far one of the most studied soluble receptors is the soluble form of the epidermal growth factor receptor (sEGFR). Growth factors like the epidermal growth factor (EGF) play an important role in promoting cancer development and progression. For their activity in restraining the growth factor signaling, the soluble receptors have been regarded for a long time as promising molecule candidates for anti-tumor treatment. However, in spite of the remarkable efforts in the understanding of mechanisms around the soluble receptors, their role in the context of cancer biology still remains unclear. The focus of this review is to elucidate the function of circulating sEGFRs, to understand which cells are responsible for their production, and to speculate on their potential use (as cancer biomarkers) in the clinical setting.

## 2. The Origin of the EGFR Soluble Counterpart (sEGFR)

**Figure 1:**
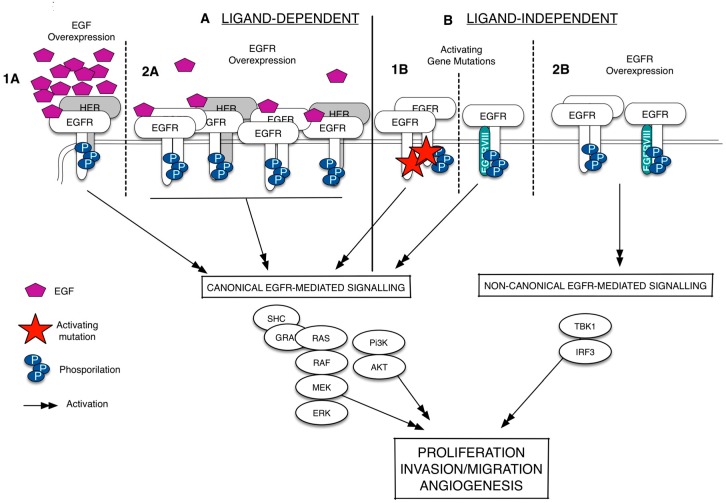
Epidermal growth factor receptor (EGFR) signaling deregulation in cancer. The EGFR signaling can be deregulated in a ligand-dependent (**A**) or ligand-independent manner (**B**). The ligand-specific activation of the wild-type epidermal growth factor receptor (EGFRwt), which occurs via EGF overexpression (**1A**) or EGFR overexpression (**1B**), leads to the EGFR homodimerization/heterodimerization, phosphorylation of specific tyrosine residues and recruitment of several proteins at the intracellular portion of the receptors. It activates a series of signal transduction pathways (black arrows), including the canonical signals as such as ERK and Akt. The ligand-independent activation happens when the EGFR shows activating gene mutations. These mutations can occur in the EGFR intracellular domain or in the EGFR extracellular domain to form a constitutively active protein (EGFRvIII) (**1B**). Without ligand, the EGFRwt overexpression can also lead to the EGFR phosphorylation and the activation of a non-canonical form of signaling that results in the activation of the transcription factor IRF3 (**2B**). Dash lines separates the different mechanisms of EGFR activation.

Since its discovery more than 20 years ago, the epidermal growth factor receptor (EGFR/HER1) has emerged as a key player in regulating cell proliferation and survival in different types of cancer [3,4,5,6]. The EGFR is a membrane-bound tyrosine kinase glycoprotein widely expressed on the surface of multiple cell types, including epithelial, mesenchymal, and neuronal cells [7]. It is composed of an extracellular domain (ECD), a lipophilic trans-membrane segment, and an intra-cellular region (ICD) containing a tyrosine kinase domain [8]. The EGFR is activated by binding to growth factors of the EGF-family (*i.e.*, the EGF and the TGFα), which are produced by the same cells that express the receptor (autocrine secretion) or by surrounding cells (paracrine secretion) [9,10]. Upon the binding of the ligand, the receptor dimerizes, either as a homodimer or as a heterodimer, preferentially with the HER2, but also with other members of the HER family; it therefore undergoes auto-phosphorylation at specific tyrosine residues within the intracellular domain that leads, in turn, to the activation of downstream signaling pathways, including the Ras/Raf/mitogen-activated protein kinase (MAPK) and PI3K-AKT ones [11]. The aberrant activation of the EGFR is strictly related with cancer development and progression [11,12]. As summarized in Figure 1, the EGFR is deregulated, in several types of cancer, by three fundamental mechanisms: (i) activating gene mutations; (ii) overexpression of the EGFR trans-membrane form (via gene amplification or polysomy) and (iii) altered ligand expression (with the possible formation of autocrine loops) [13]. In particular, the existence of mutations in the ICD of EGFR that causes a constitutive or ligand-independent EGFR signaling activation has been widely studied in lung cancer and glioblastoma [14,15]. In non-small cell lung cancer (NSCLC), these mutations shows a prevalence in Western and Asian patients and are detected mainly in adenocarcinomas [16]. Exon 19 deletion and L858R point mutation in exon 21 are the most frequently detected and together account for 90% circa of all EGFR activating mutations [17]. Recently, a non-canonical form of EGFR-mediated signaling, which does not include the activation of the usual EGFR signals as such as the ERK and the Akt, has been identified [18]. This type of EGFR-mediated signaling is ligand-independent, occurs when EGFR is wild-type (EGFRwt) and overexpressed, and results in the activation of the transcription factor IF3 [19] (Figure 1). In glioblastoma, the EGFRwt overexpression is frequently associated with a mutated EGFR, labeled EGFRvIII, resulting from a rearrangement of the *EGFR* gene [20,21]. EGFRvIII is a 140-kDa EGFR trans-membrane isoform with a truncated extracellular domain, containing an in-frame deletion of amino acids 6-273, and it is originated by a tumor specific-mutation produced by the deletion of exons 2–7 [21,22]. The G protein-coupled receptor (GPCR) agonists (such as lysophosphatidic acid, thrombin, endothelin-1, and angiotensin II) can also promote the EGFR signaling via *transactivation*, a biological process naturally occurring in both cancer and cardiovascular diseases [23,24,25,26,27]. Besides the transmembrane EGFR form, there is evidence of the existence of truncated/soluble EGFR isoforms (sEGFRs) containing the EGF-binding site but lacking the membrane-spanning region and the kinase domain. This adds another level of complexity in the definition on how the fine regulated-EGFR network works, suggesting that these isoforms may act as EGFR homeostasis regulators. As well, two mechanisms involved in their generation were characterized: (i) the alternative splicing of the full-length receptor mRNA that gives rise to a protein isoform with a shorter COOH-terminal sequence and lacking the intracellular domain; and (ii) the site-specific proteolysis (shedding) that involves the cleavage of the full-length receptor to release the extracellular domain.

### 2.1. sEGFRs Derived from EGFR mRNA Alternative Splicing

Alternative splicing is a mechanism that produces more than one mRNA transcript from the same mRNA precursor. This event is due to variations in the incorporation of the exons, a process that generates functionally different proteins [28]. The existence of the alternative EGFR mRNA transcripts hail from 1984, when Xu and colleagues discovered for the first time that the EGFR was encoded by a single gene, from which two mRNA transcripts of 10.5 and 5.8 kb were expressed [29]. The relative expression levels of these transcripts were different among tissues of origin [30,31]. In addition, the alternative transcripts encoding for the aberrant forms of EGFR were described both in tumor cell lines as well as in normal tissues. Indeed, a 2.8-kb transcript encoding for a 100-kDa sEGFR isoform was shown to be expressed in the human A431 cell line [32,33,34,35], while two mRNA transcripts of 1.8 and 3.0 kb were identified in the human placenta and the liver [36,37,38]. Reiter and colleagues [38] analyzed 17 adult tissues and 4 fetal ones, demonstrating that the human 3.0-kb EGFR mRNAs encoding for a 110-kDa sEGFR was detectable only in the human placenta, which shows high EGFR expression. Noticeably, this transcript was also observed in carcinoma cell lines that show EGFR gene amplification [38].

### 2.2. sEGFRs Derived from EGFR Ectodomain Shedding

Ectodomain shedding (ES) is the proteolytic cleavage of a trans-membrane protein, resulting in the loss of the extracellular domains (ectodomains). It is a key event in many developmental and homeostatic processes as well as in cancer [39,40,41]. ES is often activated by several factors, including the activation of the protein kinase C (PKC). The main actors involved in this process are members of two different metalloprotease families: (i) matrix metalloproteases (MMPs) and (ii) metalloendopeptidases (ADAM). These proteins are membrane-anchored metalloproteases that process and shed the ectodomains of membrane-anchored growth factors, cytokines, and receptors [42,43]. The knowledge concerning the generation of sEGFRs by the proteolitic cleavage of the trans-membrane EGFR forms is limited and still a subject of some controversy. Some authors have reported that the EGFRs could be subject to limited protein shedding. In cells exposed to potent PKC activators such as phorbol esters, the cleaved soluble forms of EGFR could not be detected [44,45]. On the other hand, Sanderson *et al.* [46] and Perez-Torres *et al.* [47] demonstrated the presence of shedding-derived sEGFRs in cell-conditioned medium (CCM) of both immortalized keratinocyte cell line HaCaT and in malignant cells that expressed 7 × 10^5^ or more receptors/cell. In particular, Perez-Torres demonstrated the existence of a 110-kDa sEGFR protein that shares amino acid sequence identity with the ECD of the EGFR at the glycine residue 625. This isoform, named PI-sEGFR, was highly glycosylated (as the full-length EGFR) and it was released in the CCM by the proteolytic cleavage process triggered by the PKC activation upon cells treatment with the phorbol 12-myristate 13-acetate (PMA) [47].

## 3. The 110-kDa sEGFR Proteins

### 3.1. Biochemical Characteristics

**Figure 2:**
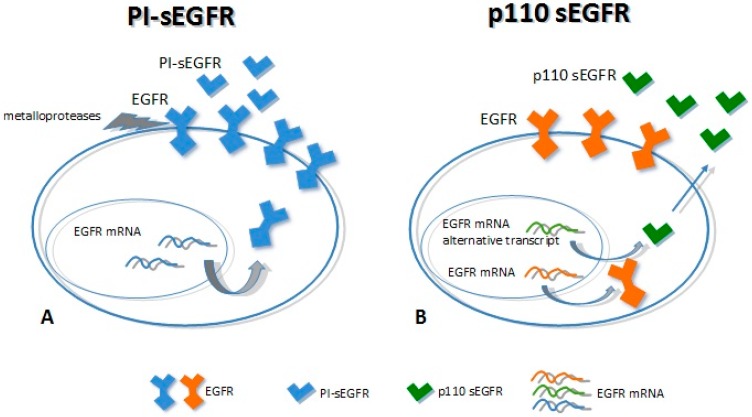
Mechanisms of soluble epidermal growth factor receptor (sEGFR) generation. (**A**) The full-length EGFR is cleaved by metallo-proteases (receptor shedding) to release the extracellular domain (PI-sEGFR); (**B**) Alternative splicing of the mRNA coding for the EGFR originates an alternative transcript that encodes (curved gray arrows) for the sEGFR p110 isoform, which is secreted in the extracellular environment (blue arrow).

Two major soluble forms of the EGFR have been characterized so far, with a molecular weight of 110 kDa each. First, p110 is transcribed from an alternative mRNA transcript of 3.0 kb [38] and is detectable mainly in healthy tissues (as the placenta). Second, PI-sEGFR derives from a proteolytic cleavage of the EGFR trans-membrane form [47], which is expressed in tumor cell lines with high EGFR expression. Even though these proteins show the same molecular weight, they have a different amino acid backbone. The p110 isoform has the same primary structure of the full-length receptor up to residue 603, thus having the same extracellular domain followed by a 78 unique COOH-terminal. The PI-sEGFR isoform has the same EGFR extracellular domain up to the amino acid 625 (Figure 2) [38,47,48]. Baron and colleagues were the first to detect a soluble form of 110 kDa circulating EGFR in human biological fluids (serum) [49]. They speculated about the origins of this sEGFR, and they showed that it corresponded to the p110 isoform (derived from the 3.0 EGFR mRNA transcript). This protein was detected in the serum of both healthy subjects and patients with ovarian cancer; noticeably, the levels of this blood circulating sEGFR were higher in healthy subjects than in patients with ovarian cancer [48,49]. Recently, we have identified two different sEGFR proteins in lung cancer tissue; these very same molecules were found to be circulating in plasma samples derived from lung cancer patients and, as well, healthy individuals. We have demonstrated that these isoforms showed the same molecular weight (110 kDa) but different biochemical characteristics. The tumor tissue showed 110-kDa sEGFR isoforms with isoelectric point (pI) >6, while plasma samples showed 110-kDa sEGFR isoforms with an extremely acidic pH (3.87–4.74), indicating that the secreted EGFR isoforms in plasma and in lung cancer were molecularly heterogeneous [50]. Moreover, we have observed that not only was the tumor-specific 110-kDa sEGFR not detectable in the lung cancer patients plasma, but also that levels of this protein were lower in lung cancer cases than in healthy subjects [50,51]. Reasonably, the 110-kDa sEGFR proteins observed in plasma and the tumor-specific ones may correspond respectively to the p110 identified by Baron and colleagues and the PI-sEGFR identified by Perez-Torres and his group [47,49].

### 3.2. Biological Functions

**Figure 3:**
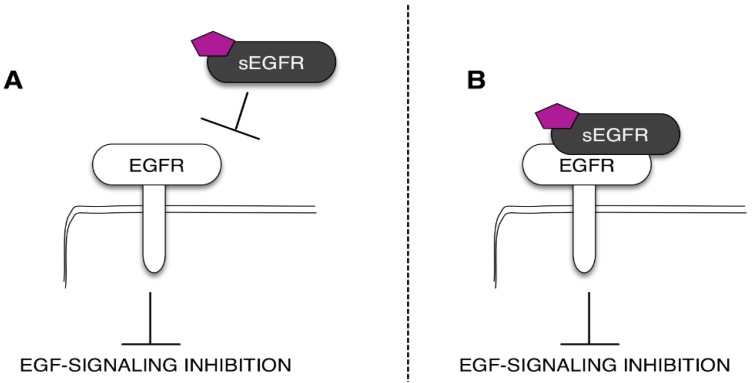
Mechanisms of the sEGFR isoform anti-tumoral action. The sEGFR can avoid the ligand-dependent EGFR activation sequestering the ligand (purple pentagon) (**A**) or directly binding the EGFR ECD (**B**).

The need to find novel molecular biomarkers to monitor the tumor development and its progression is a pivotal issue in cancer research. From a general perspective, every tumor produces signals that we should be able to recognize and use to guide the diagnostic work-out, the prognostic prevision, and, definitely, the clinical treatment of the patient. These signals are often represented by molecules involved in tumor cell growth and/or in the processes that are strictly correlated with the tumor behavior, *i.e.*, (but not limited to) the inflammation. Indeed, when we talk about a biomarker, we usually think about a molecule produced by the tumor and released in biological fluids. In this case, the “ideal” biomarker should be more abundant in cancer patients when compared with healthy subjects or patients with benign diseases. However, there are also biomarkers that we named “negative biomarkers”, which represent physiological molecules (produced by normal tissues and constitutively expressed in biological fluids) that are under-expressed in patients affected by a specific tumor. Typically, these types of biomarkers correspond to protective molecules, which are proteins able to keep off the key molecular players involved in tumor cell growth and proliferation. In light of this, the observations that sEGFR levels decrease in cancer patients support the hypothesis that these soluble forms of EGFR have a physiological and protective role against cancer. In this context, several studies indicate that sEGFRs could regulate the EGFR signaling in normal and tumor tissues [33,36,51]. Recently, we tested the potential anti-tumor effect of the recombinant sEGFR in NSCLC cell lines, demonstrating that the sEGFR is capable of inhibiting the tumor cell proliferation and the cell migration in a model without mutations in the TK domain of the EGFR [51]. Unfortunately, the mechanism underlying the sEGFR anti-tumor effect has not been fully understood. In this sense, the most common biological function hypothesized for the soluble receptors is the regulation of the EGFR activity by modulating the EGFR-mediated signal transduction. This regulation can occur in the extracellular environment through the sEGFR competition for the ligands (*i.e.*, EGF and TGFα) with the cell-surface EGFR and or through the direct interaction with the EGFR trans-membrane form (Figure 3) [52]. The ability of the sEGFR isoforms to bind the EGF family members, as well as to interact with the cell-surface EGFR has already been described [33,37,53]. Basu and colleagues [33] showed that the sEGFR may be able to interfere with the EGFR-induced signal transduction by creating inactive heterodimers with the full-length EGF. We have established that the sEGFR protein exerts its activity by interfering with the EGF-regulated protein signaling avoiding the full-length receptor internalization [33,51].

## 4. The sEGFR Potential Role in the Diagnosis and Prognosis of Cancer Patients

The existence of circulating sEGFR proteins in the bloodstream has stimulated researchers to investigate whether the amount of these molecules could be related with the presence of the tumor. Several works have explored the potential diagnostic, prognostic, and predictive roles of the sEGFR values [54,55,56,57,58] in different types of cancer. In metastatic breast cancer patients, no correlation was observed between the expression of the EGFR in the primary tumors and the sEGFR serum levels [54]; the sEGFR levels were also tested before and after the administration of trastuzumab-based therapy, but they failed to predict the efficacy of such therapy in the same type of cancer [55]. In the cervical carcinoma, Oh and co-workers [56] have determined the levels of EGFR in the serum of 38 patients (invasive or recurrent carcinoma (*n* = 26) and carcinoma *in situ* (CIS; *n* = 12)) and 38 healthy females as controls, using an enzyme-linked immunosorbent assay (ELISA). The mean serum level for sEGFR in the patients with invasive or recurrent carcinoma (165 ± 60 fmol/mL) was significantly higher (*p* < 0.0001) when compared to that measured in healthy controls (66 ± 17 fmol/mL) and was higher (*p* = 0.015) than that of patients with CIS (126 ± 25 fmol/mL). In addition, there was a significant difference in the mean serum levels of sEGFR between patients with CIS and healthy controls (*p* < 0.0001). The authors assumed that the detected sEGFR was the product of a proteolytic cleavage of the full-length receptor deriving from the tumor and suggested a potential usefulness of the sEGFR as a biological marker of cervical carcinoma. Choi and colleagues [57], who tested the sEGFR values in a dataset of gastric carcinoma patients, reported similar results. They had found significantly elevated sEGFR values in cancer patients compared to the healthy population; furthermore, the levels change according to the tumor progression activity and its response to therapy. These observations allowed for the proposal of the sEGFR as a reliable tumor marker of gastric carcinoma for diagnosis, prognosis, surgery follow-up, and chemotherapy efficacy. Partanen *et al.* [58] examined the banked serum samples of 38 asbestosis patients who developed cancer, matching the sEGFR values with a population of 72 asbestosis patients without cancer, and 20 healthy controls. They observed significantly higher sEGFR values in the first group of cases compared to the other ones, concluding that the soluble isoform of the EGFR may be elevated in the early stages of carcinogenesis in asbestosis patients. On the other hand, other evidence supports the concept that circulating sEGFR may be a physiological molecule with a tumor-protective role since it seems to be elevated in healthy subjects and tends to decrease in patients with neoplastic lesions [49,59,60]. These studies investigated whether the alterations in sEGFR levels may be useful in cancer diagnosis, therapy response, and monitoring disease recurrence and outcome. A decrease in the sEGFR levels in cancer patients was observed especially in patients with breast, ovarian, and lung cancer [59]. Concerning the study of the diagnostic potential of the sEGFR, its decrease was observed in several types of cancer: (a) Breast. Asgeirsson and colleagues [60] demonstrated that sEGFR levels were significantly higher in normal individuals (median 75.3 ng/mL, range 43.2 to 114.2 ng/mL) than in patients with primary breast cancer (median 59.3 ng/mL, range 21.3 to 94.1 ng/mL). Moreover, the sEGFR levels were even lower when comparing the sEGFR values at the time of primary diagnosis (median 56.3 ng/mL, range 29.1 to 142.7 ng/mL) and those at the time of distant relapse (median 30.9 ng/mL, range 10.9 to 106.4 ng/mL) [60]; (b) Ovarian. Baron and colleagues were the pioneers in the sEGFR quantification [49]. Ovarian cancer is the first neoplasia in which the sEGFR levels were studied in order to identify a circulating molecule able to discriminate between cancer patients and healthy subjects. In this context, it was noted that patients with epithelial ovarian cancer had lower circulating sEGFR levels than women with either benign ovarian neoplasms or benign gynecologic conditions of non-ovarian origin, suggesting its potential role as a biomarker in such tumors. Moreover, the sEGFR concentrations do not differ between women with benign ovarian neoplasms and benign non-ovarian gynecologic conditions [49]; (c) Lung. As a potential biomarker in the diagnosis of NSCLC, several groups (Table 1) demonstrated a decrease in sEGFR levels in patients affected by primary lung tumors. In 2007, Lemos-González and colleagues [61] observed that the sEGFR in the serum of a healthy subject fitted a normal distribution in which the mean level of this protein was 35.9 ± 5.2 ng/mL, while NSCLC patients showed a sEGFR mean concentration of 25.5 ± 4.5 ng/mL [61]. Our group also investigated the diagnostic performance of the sEGFR in the last decade. A significant reduction of circulating sEGFR was detected in the NSCLC patient group (34.59 ng/mL; range: 26.74–47.38) as compared with the control group (46.40 ng/mL; range: 41.71–54.00). Recently, we performed a further analysis comparing the plasmatic sEGFR levels of a heterogeneous cohort of 37 non-advanced NSCLC patients with those coming from 54 healthy subjects by using the ELISA methodology [51]. In line with our previous results [50], we found that the plasma sEGFR significantly decreased in the NSCLC patient group as compared to the control group (median value: 48.6 *vs*. 55.6 ng/mL respectively; *p* = 0.0002). Moreover, we concluded a long-term clinical-radiological surveillance of the entire NSCLC population, and we explored the potential role of the sEGFR as prognostic factor. Although we observed a slight trend, we did not show any statistically significant prognostic difference according to the sEGFR values when adjusting the sample for sex, age, and smoking habits. In line with our data, Jantus-Lewintre and colleagues [62] reported, in a large cohort of advanced NSCLC patients (308 pts with Stage IIIB/IV disease), a significant association between the sEGFR value and prognosis, with the lower baseline sEGFR values significantly associated with a reduced survival. Finally, the sEGFR was studied also regarding its potential role as a biomarker for the prediction of treatment response and to monitor the patients follow up. Two research groups have observed, independently, that high circulating sEGFR levels were predictive of an increased response rate to both metronomic chemotherapy and gefitinib treatment [63,64].

## 5. Conclusions

The sEGFR is a soluble isoform of the EGFR, composed only by the extra-cellular domain (ECD) of this protein, which can be measured directly in the biological fluids like serum/plasma. This protein seems to have a good potential as a biomarker, but its overall role in cancer is not yet well defined. Few authors [56,57] claim that the sEGFR could be a traditional “positive biomarker” released in biological fluids by tumor cells that normally express high levels of the EGFR. On the other hand, other authors [61,62] consider the sEGFR as a physiological molecule with a protective role in the control of the EGFR-regulated signaling. Concerning NSCLC only, there are emerging data suggesting that such tumors produce a specific sEGFR isoform that has specific biochemical characteristics and, apparently, is not detectable in plasma samples of NSCLC patients [50]. Conversely, plasma deriving from NSCLC patients shows another isoform of the sEGFR, with different biochemical properties. This isoform was detectable also in healthy subjects, leading us to hypothesize that what researchers quantified in plasma may be a physiological sEGFR molecule*. In vitro* experimental studies, analyzing the potential anti-tumor activity of recombinant sEGFR on lung cancer cell lines, have reported a decrease in lung tumor cell growth and migration, suggesting that this protein could have an anti-proliferative effect. Few studies have evaluated the role of sEGFR in NSCLC patients, with a dual aim of detecting its potential as a circulating tumor biomarker and evaluating its prognostic role. Most clinical experiences [61,62] suggest that the sEGFR is a circulating EGFR isoform with a protective role (negative biomarker) in NSCLC, representing a potential biomarker in this form of cancer early-detection. If we deem this assumption well evidence-supported, we can hypothesize a potential use of this biomarker in the context of primary screening programs. Indeed, its overall potency should be matched, for example, with other forms of screening, where, for example, the recent results of the National Lung Screening Trial (NLST) have demonstrated a reduction in the lung-cancer mortality with low-dose computed tomographic screening [65]. The NLST Research Team stated that molecular markers in blood, sputum, and urine one day will help to select persons who are best suited for low-dose CT screening or to identify persons with positive low-dose CT screening tests who should undergo more rigorous diagnostic evaluation, thus repositioning the value of putative biomarkers as the sEGFR to a (sub)-stratifier in strategies based on imaging techniques. As well, innovative systems-biology/systems-medicine based methodologies, as the one proposed in [66], should be taken into consideration when discussing lung cancer screening programs. In fact, in this setting, the value of bronchogenic “genomic” biomarkers has been recently under intense scrutiny [67,68,69,70]. Considering that plasma samples are readily available in NSCLC patients, and by coupling the facts that this type of analytical test is affordable and with a high potential to provide valuable prognostic information, it would indeed be worth evaluating the sEGFR in a prospective large-scale articulated screening program study, evaluating it as a potential cancer biomarker for the early diagnosis of NSCLC. Finally, the data regarding the potential of sEGFR as a prognostic biomarker in patients with various solid tumors are very controversial. Although some studies reported a significant association between sEGFR value and prognosis, with lower baseline sEGFR levels associated with reduced survival, to date there are no robust data supporting its clinical use as a prognostic classifier in NSCLC.

## Figures and Tables

**Table 1:** Overview of the relevant literature concerning the role of sEGFR in NSCLC.

Researcher	Population	sEGFR Detection Method	Main Findings
Lemos-Gonzalez 2007 [61]	25 NSCLC *vs.* 50 healthy donors	ELISA (R&D system)	Significant lower sEGFR values in NSCLC-patients.
Jantus-Lewintre 2011 [62]	308 NSCLC *vs.* 109 healthy donors	ELISA (R&D system)	Significant lower sEGFR values in NSCLC-patients.
			sEGFR as a significant independent prognostic marker.
Maramotti 2012 [50]	12 NSCLC *vs.* 12 healthy donors	ELISA (Ray Boitech)	Significant lower sEGFR values in NSCLC-patients.
Lococo 2015 [51]	37 not-advanced NSCLC *vs.* 54 healthy donors	ELISA (R&D system)	sEGFR inhibits growth and migration of NSCLC cells *in vitro.*
			Significant lower sEGFR values in NSCLC-patients.

## Abbreviations

sGFRs	soluble receptors
EGFR	Epidermal Growth Factor Receptor
sEGFR	soluble epidermal growth factor receptor
MMPs	matrix metalloproteases
CCM	cell conditioned medium
NSCLC	non-small cell lung cancer
CIS	carcinoma *in situ*
